# Dengue Vector Control through Community Empowerment: Lessons Learned from a Community-Based Study in Yogyakarta, Indonesia

**DOI:** 10.3390/ijerph16061013

**Published:** 2019-03-20

**Authors:** Sulistyawati Sulistyawati, Fardhiasih Dwi Astuti, Sitti Rahmah Umniyati, Tri Baskoro Tunggul Satoto, Lutfan Lazuardi, Maria Nilsson, Joacim Rocklov, Camilla Andersson, Åsa Holmner

**Affiliations:** 1 Department of Epidemiology and Global Health, Umeå University, 90187 Umeå, Sweden; maria.nilsson@umu.se (M.N.); camilla.andersson@umu.se (C.A.); Asa.Holmner@regionvasterbotten.se (Å.H.); 2 Department of Public Health, Universitas Ahmad Dahlan, Yogyakarta 55164, Indonesia; fardhiasih.dwiastuti@ikm.uad.ac.id; 3 Department of Parasitology, Gadjah Mada University, Yogyakarta 55281, Indonesia; sittirahmahumniati@yahoo.com (S.R.U.); tribaskorots2@gmail.com (T.B.T.S.); 4 Department of Health Policy and Management, Gadjah Mada University, Yogyakarta 55281, Indonesia; lutfan.lazuardi@gmail.com; 5 Department of Public Health and Clinical Medicine, Umeå University, 90187 Umeå, Sweden; joacim.rocklov@umu.se; 6 Department of Radiation Sciences, Umeå University, 90187 Umeå, Sweden

**Keywords:** dengue, community participation, empowerment, vector control

## Abstract

Effort to control dengue transmission requires community participation to ensure its sustainability. We carried out a knowledge attitude and practice (KAP) survey of dengue prevention to inform the design of a vector control intervention. A cross-sectional survey was conducted in June–August 2014 among 521 households in two villages of Yogyakarta, Indonesia. Demographic characteristics and KAP questions were asked using a self-managed questionnaire. Knowledge, attitudes and practice scores were summarized for the population according to sex, age, occupation and education. The average knowledge score was rather poor—3.7 out of 8—although both attitude and practice scores were good: 25.5 out of 32 and 9.2 out of 11 respectively. The best knowledge within the different groups were found among women, the age group 30–44 years, people with a university degree and government employees. Best practice scores were found among retired people and housewives. There were several significant gaps in knowledge with respect to basic dengue symptoms, preventive practices and biting and breeding habits of the *Aedes* mosquito. In contrast, people’s practices were considered good, although many respondents failed to recognize outdoor containers as mosquito breeding sites. Accordingly, we developed a vector control card to support people’s container cleaning practices. The card was assessed for eight consecutive weeks in 2015, with pre-post larvae positive houses and containers as primary outcome measures. The use of control cards reached a low engagement of the community. Despite ongoing campaigns aiming to engage the community in dengue prevention, knowledge levels were meagre and adherence to taught routines poor in many societal groups. To increase motivation levels, bottom-up strategies are needed to involve all community members in dengue control, not only those that already comply with best practices.

## 1. Introduction

Dengue fever, one of the most significant mosquito-borne diseases worldwide, is becoming an increasing threat to humanity owing to climate change and globalization [1,2,3,4]. Indonesia is no exception. There have been several outbreaks historically [5] and since the late 1960s, the number of cases has increased approximately 2000 fold [6]. Dengue fever is currently infecting people in all 34 districts of this large island nation [7] and Yogyakarta City, the location of the present study has become a dengue hotspot in recent years. In 2014, the average Incidence Rate (IR) in Yogyakarta City was 101.10 per 100,000 population [8], which was twice the national average of 50.75 [9]. In 2016, the number of reported cases reached 1706 [10], corresponding to an IR of 413.74 per 100,000 population, which was more than five times the national average of 77.96 per 100,000 population [11]. So far in 2019, 35 cases of dengue fever have been reported in Yogyakarta according to the District Health Office in January, a fivefold increase compared to the same period in 2018 [12]. Furthermore, the incidence rate is likely to be even higher as dengue is severely underreported globally according to the World Health Organization [13].

There is a general consensus that no single intervention will be sufficient to combat dengue fever [14]. It is foreseen that an integrated approach consisting of evidence-based strategies proven effective and appropriate for local conditions will be required [15,16]. This includes community-based strategies, such as education and cleaning programs aiming to eliminate the number of mosquito breeding sites inside and nearby people’s homes [17,18]. Community involvement in vector control has been reported effective [18], although the evidence remains inconclusive. Many barriers have been identified that might influence the success and, above all, the sustainability of community-based initiatives [19]. 

In Indonesia, dengue programs build vertically from national to provincial level, district and sub-district levels and incorporate epidemiological surveillance, vector control, public health campaigns as well as education, training and research [20]. In 1992 the Ministry of Health initiated the so-called 3M program, where the three Ms stands for Menutup, Menguras and Mengubur, meaning covering of water containers, cleaning of water containers and burying of discarded containers. This was later followed by the 3M plus, which included specific activities aiming to reduce mosquito breeding places and education on protective behaviour. The one key factor in these strategies was community involvement [6] which included health education through mass media, implementation of the programmes through door-to-door visits from village volunteers and source reduction of larvae through community participation and inter-sectoral coordination [21]. These programs have been implemented throughout Indonesia under the coordination of local health authorities; however, although well established, program evaluations are lacking, and the extent to which citizens are acting according to these principles in Yogyakarta is still unknown. 

This study aimed to assess the current status of people’s knowledge, attitudes and practices with regards to dengue fever prevention as a baseline to inform the development of a vector control intervention in the local community. The study was performed in close collaboration with local health workers and the local health authority. Here we report the results from a so-called KAP (knowledge attitude and practice) questionnaire addressing people’s knowledge, attitudes and practices and the result from a feasibility study addressing the potential of using control cards as a stand-alone intervention to reinforce people’s container cleaning practices. 

## 2. Materials and Methods

### 2.1. Study Design

**Figure 1:**
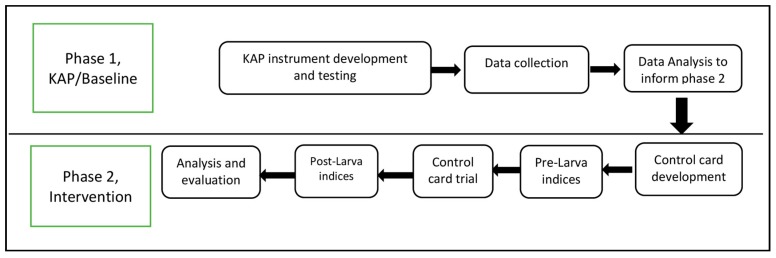
Schematic illustration of the study design.

Two urban-villages in Yogyakarta were included in the study, Mantrijeron and Demangan. These villages were selected as they at the time had comparable dengue incidence rates (IR); 30.9 and 30.0 per 100,000 population in Mantrijeron and Demangan respectively, according to the City Health Office data for 2014. The study was executed in two phases during 2014–2015: The first phase, a cross-sectional KAP survey was conducted in both villages during 2014. Information from this study, particularly responses to questions with a low percentage of correct answers was used to guide the design of the intervention to be executed in the second phase. In this phase, control cards for self-monitoring of container cleaning practices were designed and distributed to households in one of the villages (Mantrijeron) and monitored with support from local field workers. The control was intervened as usual. The cleaning routines were monitored for eight consecutive weeks in late spring 2015. To evaluate the feasibility of using control cards as a standalone intervention to improve people’s cleaning practices, Demangan was used as a control during this part of the study. Information about the number of houses infested with larvae and the corresponding number of larvae positive water-holding containers in each house were collected in both the intervention and the control site before and after the study. At the end of the control card study, a short survey was conducted among the team of field workers monitoring the trial to assess their experiences and opinions about the control cards and their feasibility as a standalone intervention. Research flow is presented in Figure 1.

### 2.2. Study Instruments

#### 2.2.1. Baseline KAP Survey

The instrument consisted of 29 questions grouped into four main categories: (1) socio-demographics of the respondent (sex, age, education, occupation); (2) knowledge (disease, agents, symptoms, transmission and treatment); (3) attitudes (who is at risk, importance of container cleaning, seriousness of the disease) and (4) precautionary practices (container cleaning, protective behaviour). The knowledge and practice questions were designed with multiple-choice items, whereas attitudes were assessed using statements and a 4-level Likert scale ranging from “strongly disagree” to “strongly agree”, in addition to the optional answer “I don’t know” which was scored 0 points. Correct answers on knowledge and practice questions were scored 1 point, except for one especially important practice question addressing container cleaning practices. This question was scored 0–3; with 3 representing the best practices and 0 the worst practices. Maximum knowledge and practice scores were 8 and 11, respectively. Attitudes were scored 0–4, with 4 representing the most proper attitude and 1 the worst attitude. A complete lack of answers scored 0 points. The maximum score for attitude was 32. 

Prior to the study, the KAP instrument was tested for reliability on 30 people in Umbulharjo, a sub-district not included in the study. Cronbach’s Alpha was 0.6 for Knowledge and 0.8 for both Attitude and Practices [22], which was deemed sufficient for our purpose. The results from the baseline survey were thereafter analysed, particularly with respect to weaknesses in peoples replies, and used to inform the design of the intervention in phase two.

#### 2.2.2. Control Cards and Monitoring Forms

Based on the outcome of Phase 1, control cards focusing on domestic chores important for dengue control was chosen as an intervention to improve awareness and support individual households in their dengue preventive activities. The cards were designed based on literature reviews and discussions with the local health authority in charge of the community-based vector control initiatives. The design was kept simple to allow various community members to use them, regardless of age and literacy level. The card displayed one field for entering the household identity and additional fields for the users to record all types of containers that could store water in and near their household, for example “bak mandi” (traditional tub/large container in the bathroom), flower vases, pots and buckets, and for putting a mark (√) once the containers were cleaned. Households included in the trial were recommended to clean their surroundings and monitor the procedure using the cards at least twice a week. Field workers regularly monitored the study through random visits and recorded on site the cleaning activities performed using a custom-made monitoring form. These forms included the names of the households visited, whether they had performed any container cleaning and the type of containers cleaned. The same groups were visited for pre- and post-monitoring. The opinions of the field workers were assessed through a short questionnaire distributed approximately one week after the feasibility study had ended. The questionnaire included open questions addressing whether the field workers distributed the cards; whether they perceived the cards useful, and finally, whether the households used the cards or not together with their arguments why/why not. 

### 2.3. Sample and Procedure

#### 2.3.1. Baseline Survey

Based on local village government records, there were 2440 and 3392 households in the villages Mantrijeron and Demangan, respectively. A sample was calculated by considering a 90% confidence level, a ±10% margin for error and a response distribution of 50%. Accordingly, the minimum sample size was 244 for Mantrijeron and 251 for Demangan. We deliberated incomplete data by adding another 4% of households. Accordingly, the baseline survey was executed in 257 households in Mantrijeron and 264 households in Demangan. Each household was represented by one person and all questionnaires were completed on the first visit. The participant’s inclusion criteria were (1) adults, ≥15 years of age with the ability to communicate orally and in writing and (2) willing to participate in the study. People who had lived in the village less than one year were excluded from the survey. Because of limited resources, convenient sampling was applied to select the households for the study. Door-to-door visits were carried out to invite households to participate. If no one was at home, or if the household chose not to participate, the next household was chosen and family members who met the inclusion criteria were recruited. In case several family members were present, the head of the family (typically the husband according to Indonesian tradition) was selected to participate in the survey, even though traditionally women are in charge of domestic chores. If the head of the family was absent, the wife or another family member who met the inclusion criteria was chosen to replace him/her. After confirming that a participant met the inclusion criteria, the questionnaire was distributed, and informed consent was obtained. Questions were distributed in Bahasa and translated to English at the end of the study. 

#### 2.3.2. Pre-Post Intervention

The control card feasibility study started on the first of June 2014 and ran for eight consecutive weeks in the two villages. Before the study, 76 voluntary field workers representing different geographical sub-areas (called RW) within the village were informed on how to monitor the study. Thereafter they distributed the control cards to households in their respective RW. In total, 2440 control cards were circulated, approximately 30 cards per sub-area. The container cleaning practices were regularly monitored by the field workers, who checked water containers and the usage of the cards through random visits to ten households on a weekly basis. During the second month, the monitoring was executed every second week. Each monitoring round addressed 760 households in total. 

At the end of the trial, the field workers opinions about the study were assessed using a simple questionnaire. The questionnaire aimed at assessing how the field workers perceived the effectiveness of the control cards, and their views on the community’s acceptance of the intervention. Questionnaires were analysed with respect to its qualitative content. 

To evaluate if the control cards enhanced peoples cleaning practices, the number of larvae infested houses and containers were assessed in both villages pre and post the intervention. Minimum sample sizes were estimated to 141 households in Mantrijeron and 143 households in Demangan, based on an 80% statistical power, 5% confidence limit and an expected frequency of positive larvae of 35% (Statcalc Epi Info 7). 

#### 2.3.3. Statistical Analysis

The Statistical Package for Social Sciences (SPSS) version 24.0 (IBM, Armonk, NY, USA) was employed for all statistical analysis. The baseline survey was analysed by descriptive and univariate analysis. The outcome of the feasibility study was evaluated by comparing the number of houses and containers infested with larvae between the intervention and the control group pre and post the intervention using a Poisson regression model. This analysis used time parameters (pre/post) and group parameters (intervention/control) to estimate the change over the study period by interacting time with the group variable longitudinally. 

### 2.4. Ethical Considerations

The study was approved by the Ethical Review Board of Universitas Ahmad Dahlan, Yogyakarta, Indonesia (ethical approval code: 011509070). Research permission was in addition obtained from the City Health Office and the heads of the two sub-districts involved in the study. Written and/or oral consent was acquired from the head of all households participating in the study, or from another family member ≥15-years of age. Participants were informed that they had the right to withdraw from the study at any time and for any reason.

## 3. Results

### 3.1. Baseline Assessment through KAP Survey

In total, 521 individuals completed the KAP baseline survey from the two villages. All who were invited chose to participate. Most participants were in the age group 45–59 years old (34.5%), and a majority (59.7%) had graduated from secondary school. Many of the participants (36.1%) worked in the private sector. The two research areas were comparable with respect to socio-demographic determinants: age, education, occupation and sex. In addition, the two groups had comparable knowledge, attitude and practice scores. Participant characteristics and mean scores are summarized in Table 1. 

#### 3.1.1. Knowledge

Mean knowledge score for the total sample was 3.7 (SD = 1.6) out of 8. Females had a better knowledge (mean = 3.8, SD = 1.7) compared to the men (mean = 3.6, SD = 1.5) and people in the age group 30–44 years had better knowledge (mean = 4.2, SD = 1.6) compared to other age groups. Government employees presented better knowledge than other groups (mean = 4, SD = 1.2). Respondent with a university degree had better knowledge than other education groups (mean = 4.3, SD = 1.5) (Table 1). 

While looking into specific questions, the survey revealed poor knowledge levels on several topics relevant to effective dengue control. Many of the respondents appeared to have limited knowledge about the habits of *Aedes* mosquitoes; only 27.4% answered correctly that one way to prevent dengue is to use repellents in the morning till evening, 37.4%, answered correctly that the mosquito is day active, biting primarily in morning and evenings. More than half of the respondents were aware of mosquitoes breeding inside their house, although their knowledge about outdoor breeding habitats was poor. Less than 30% of respondents answered correctly that ditches outside their house are not possible *Aedes* breeding sites. Poor knowledge was similarly found regarding symptoms and signs of dengue. Most respondents (>80%) knew dengue cannot be transmitted from person to person. However, less than one third (27.4%) recognized continuous high fever as a probable symptom of dengue fever (Table 2). 

#### 3.1.2. Attitude

Overall, the study population had what can be defined as good attitudes with respect to dengue fever and disease prevention with a mean score of 25.5 (SD = 4.0) out of 32. Females had a higher attitude score (mean = 26.1, SD = 2.9) compared to the men (mean = 25.1, SD = 4.1). People in the age group 30–44 years had the best attitude score (mean = 26.7, SD = 3.8) compared to other age groups. People who graduated from university had a better attitude than other educational groups (mean = 26.6, SD = 3.2). Government employees presented the best attitude score among the occupational groups (mean = 27.6, SD = 2.7) (Table 1).

When looking at specific questions, more than 40% of the respondents disagreed or strongly disagreed to omit water container with larvae inside. 63.3% of the respondents disagreed with the statement that it is not necessary to clean the bathtub, if not being dirty. More than half of the respondents stated that it is necessary to brush the containers to eliminate the mosquito eggs. 57.0% of the participants disagreed with the statement that it is acceptable to neglect discarded material such as cans and bottles outside the house. More than 60% of participants said that it is necessary to monitor larvae also outside the house. Regarding dengue transmission, most of the respondents agreed that everyone has an equal possibility to be infected by dengue fever. Finally, slightly more than half (52.6%) of the participants strongly agreed that it is necessary to take people with suspected dengue to the hospital immediately (Table 3).

#### 3.1.3. Practice

The study population was found to have good dengue prevention practices overall with a mean score of 9.2 (SD = 1.3) out of 11. Women had a better practice score than men with a mean of 9.4 (SD = 1.1) and 8.9 (SD = 1.4) respectively. Respondents aged 30–44 years had the best practice (mean = 9.4, SD = 1.2) compared to other age groups. People with a university degree had better practice scores than the other educational groups (mean = 9.2, SD = 1.4). Retired people and housewives were found to have the best dengue preventive practice overall (mean = 9.4, SD = 0.9) (Table 1).

When looking at individual questions, the study population appeared to comply well with best practices in community-based vector control. More than 90% of the respondents reported that they pay attention to the presence of larvae in water containers, that they clean and brushed water containers if any larvae are found inside and that they cleaned containers one to three times a week. 64.9% reported that indoor water containers are kept closed if possible and almost 97.9% of the respondents reported that they covered or recycled unused cans or bottles outside the house. Questions regarding adherence to proper protective practices generated a different result. More than 60% of the participants answered that they do use mosquito repellents, although less than 20% reported that they use repellents, mosquito coil or mosquito spray in the morning and evening, which might imply poor awareness of the biting habits of the vector. (Table 4). 

### 3.2. Control Card Feasibility Study

The feasibility study ran between June and August 2015 in Mantrijeron, with Demangan as a control. In total 2440 control cards were distributed, 30 cards per RW. 46 of the 76 field workers (60.5%) who were engaged in the study completed the evaluation questionnaire. According to this evaluation, 95.7% answered that they distributed the control cards in the community. 78.3% answered that the cards did help the community with larvae monitoring and 82.6% answered that the cards helped themselves with their task. On the question of whether the community used the cards, 28.3% answered “yes, a lot”, 63.0% “yes, a little”, and 6.5% answered “no”. In contrast, about 59% of the field workers said that there were difficulties in getting people to use the control cards in the intended way. A qualitative thematic analysis of the free text replies revealed several reasons for not using the cards of which the most common answers were lack of time to fill in the cards; that they were busy with their work and that they wanted the containers to be monitored by the field workers, not by themselves. In addition, as many as 61.5% of the field workers wrote that there was a lack of willingness to fill in the cards. Moreover, many households chose to fill in the cards during the field worker’s visit. Based on the cards collected by the field workers indoor bathtubs, water reservoirs, flower vases, outdoor ditches and metal barrels were the most frequently cleaned containers. 

The number of houses infested with larvae before the intervention were 28 out of 141 in the intervention site and 36 out of 143 in the control site. At the end of the intervention, these numbers had changed to 30 and 27, respectively. Therefore, the number of larvae positive houses had decreased in the control site and increased in the intervention site. The containers checked within each household varied between the pre- and post assessment as many potential breeding sites are temporary, for example, flower pots and vases. Hence, the number of containers infested with larvae were 28 out of 252 in the intervention site before the study and 34 out of 290 after the study. In the control site the corresponding numbers were 48 out of 361, and 32 out of 391, pre and post the intervention respectively. Taking into consideration the pre-post intervention-control design of the study, we conducted a Poisson regression analysis to identify the effect of the intervention. As shown in Table 5, the number of containers infested with larvae increased 1.71 times in the intervention group compared to the control group (*p*-value: 0.11; 95% CI: 0.87–3.36). Correspondingly, we found that the number of larvae positive house increased 1.42 times in the intervention group (*p*-value: 1.42; 95% CI: 0.69–2.92). However, the changes were overall not statistically significant.

## 4. Discussion

To involve the community in vector control has been proven effective [23] but there are also multiple potential barriers identified that can influence the outcomes, as described by Heinze et al. in a recent review [24]. As dengue cases remain high in Yogyakarta, there are reasons to believe that the vector control initiatives can be further improved, and people’s response to ongoing campaigns has according to our knowledge not been evaluated previously. 

Our study attempts to provide an understanding of the current knowledge, attitude and practices with regards to dengue fever and dengue prevention as a foundation for improved community participation in dengue control in Yogyakarta City. First, we assessed people’s knowledge, attitudes and practices with respect to dengue fever and dengue control using a so-called KAP questionnaire. Second, based on the outcome of this baseline study a control card intervention with a pre-post intervention-control design was executed to further explore people’s vector control practices and to better understand if regular monitoring of container cleaning practices on household level would lead to a decrease in local larvae indices. 

In summary, there seem to be significant gaps in the study population concerning basic dengue symptoms, prevention practices and *Aedes* biting and breeding habits, particularly with respect to outdoor breeding sites. The overall knowledge score in the study population is moderate at best (mean = 3.7, SD = 1.6). In contrast, attitude and practice scores were considered good or very good respectively (mean = 25.5, SD = 4.0 and mean = 9.2, SD = 1.3). Most of our respondents demonstrated proper attitudes and practices relating to vector control in general, and indoor cleaning in particular. Notable weaknesses were found in regard to peoples understanding of mosquito biting habits and the importance of attending also to outdoor breeding habitats. Nonetheless, the study population appeared to acknowledge their own role in dengue prevention. 

According to the control card intervention, however, only a few of the households recorded their cleaning activities using the cards, and there was no reduction in larvae positive at the end of the intervention study. In fact, the vector population had only declined in the control site, although the changes were not statistically significant. Hence, the participants may have performed cleaning activities as indicated by the proper replies in the baseline survey but failed to fill in the cards. It is also possible that the participants neither cleaned nor filled in the cards as anticipated, regardless of what was stated in the questionnaire. It is also likely that the high practice scores were overvalued as practice questions were posed as multiple-choice questions, and proper answers may be relatively easy to identify regardless of previous knowledge. In any case, it is safe to conclude that the control cards failed to positively influence people’s vector control habits, which has several possible explanations. 

First, the discrepancy between the knowledge and the practice scores implies that people might be ignorant or poorly motivated to engage in vector control, despite a good awareness of the practices that the health authority teaches. There are studies indicating that knowledge levels might directly influence dengue preventive practices [25,26]. However, many other studies have failed to establish a direct correlation between knowledge and practices [27,28,29]. Hence, we also need to look at people’s motivation, not only their knowledge levels. According to the so-called health belief model (HBM), people will not engage in healthy behaviour unless they value the outcome related to the behaviour. And, they need to believe that behaviour is likely to result in the desired outcome [30]. Seen from our context, we can hypothesize that people have learned at least some behaviours that can prevent disease, for example, that they know how to get rid of the mosquito larvae. But do they believe their cleaning practices will result in a decrease in mosquito larvae, and in the end, the decrease of dengue? Or do they feel their work is meaningless? This depends on the level of knowledge about the risk of getting dengue and how the people perceive the risk of *not* doing the labour. This is supported by a study by Wong et al. who found that people’s perception of their own susceptibility to dengue influenced their dengue preventive behaviour [26]. Peoples motivation also depends on whether they believe that the labour has any effect, for example, that regular cleaning will indeed result in a reduction in larval population and number of dengue cases. This requires feedback from the local authorities and therefore, it is important to assess whether people have received any feedback on their labour or not. If the answer is yes, how was this information delivered and was it received as intentioned?

Despite this long history of educational programs, education still seems to be needed, but it is also important to identify the trigger of people’s motivation to contribute to vector control initiatives. This is unlikely to be the same for different people or groups in the community as there are some important cultural factors at play here as well. In the Indonesian setting, women are responsible for the health of the entire family. Hence, there is a cultural norm stipulating that it is the women who engage in dengue prevention as to preserve family health. For them, cleaning is a regular task and does not necessarily imply recognizing the essence of dengue prevention. Hence, it is feasible to expect women, particularly housewives, to have the best knowledge and practices in regard to vector control, which is what was found in our and many other studies [25,31]. But to combat dengue fever, it is probably not enough for one group in society to engage in vector control, and we need to find ways to involve everyone in these tasks. 

Second, both the governmental initiatives and the intervention study presented here are top-down in character, which might have created a resistance to participate in the intervention. Surely, the community was engaged in the study, but as participants, not as co-creators. This might explain the rather low participation in the feasibility study where only about one in four used the control cards as intended. According to the field workers, there were three main reasons for not participating, of which one was that participants thought cleaning should be the responsibility of the field workers, not themselves. Previous research has indeed suggested that bottom-up approaches are much more likely to become successful and sustainable [32]. It is thus recommended that the health authority investigates the community’s opinions about ongoing dengue control programs, in addition to continuing to improve people’s knowledge and motivation to participate. 

This study had some limitations that need to be considered when interpreting the results. First, during validation of the KAP questionnaire, we settled for a Cronbach’s Alpha for knowledge that is rather low (0.6) although close to the ideal and still categorized as moderately reliable [33]. This could imply that our knowledge score is underestimated. In contrast, we might have overestimated peoples attitude and practice scores as a questionnaire that provided multiple choice answers may make it possible for participants to identify the proper choice [34], regardless of whether or not this applies to them. These potential issues are, however, not likely to significantly alter the outcome and similar patterns are reported elsewhere [30]. 

Second, the feasibility study aiming to evaluate the potential of control cards to enhance people’s container cleaning practices was only piloted for eight weeks, which is likely to be far too short to produce any noticeable effects. According to previous research, most community-based interventions have a duration of 6–12 months, or more [24]. Moreover, the control card trial was executed in early summer. Even though the pattern of dengue incidence has changed significantly in recent years, people still consider the winter months to be dengue season. Hence, vector control might not be considered a priority at this time of the year. Finally, there was limited information available regarding interventions that had been conducted in the study areas in recent years. It was recently discovered that another research project had executed an intervention in the control site Demangan a couple of years before this study, which might explain the differences in outcome, however small [35]. 

## 5. Conclusions

In conclusion, this study has contributed insights into the knowledge, attitudes and preventive practices in Yogyakarta after decades of community-based dengue interventions. Our outcomes support previous studies that suggest that people need not only knowledge but also a strong motivation to participate in vector control activities, which might be currently lacking. Although certain groups in the community seem to take their responsibility seriously, it is important to engage the whole society in dengue control. Hence, we stress the importance of implementing bottom-up strategies that involve the community in the design, execution and evaluation of any health intervention to make them sustainable. For future studies in dengue prevention, we suggest exploring how dengue information and risk communication can become more individualized to trigger the motivation and engagement of many societal groups. 

## Figures and Tables

**Table 1:** Characteristic of participants and KAP (knowledge attitude and practice) mean score.

Socio-Demographic	*N* (%)	Mean Score		
		Knowledge ± SD	Attitude ± SD	Practice ± SD
All participants	521 (100)	3.7 ± 1.6	25.5 ± 4.0	9.2 ± 1.3
Sub-district				
Mantrijeron	257 (49.3)	3.7 ± 1.6	25.8 ± 3.4	9.2 ± 1.2
Demangan	264 (50.7)	3.7 ± 1.5	25.5 ± 3.7	9.2 ± 1.3
Sex				
Male	234 (44.9)	3.6 ± 1.5	25.1 ± 4.1	8.9 ± 1.4
Female	287 (55.1)	3.8 ± 1.7	26.1 ± 2.9	9.4 ± 1.1
Age group (years)				
15–29	71 (13.6)	3.9 ± 1.4	26.5 ± 2.7	9.0 ± 1.3
30–44	142 (27.3)	4.2 ± 1.6	26.7 ± 3.8	9.4 ± 1.2
45–59	180 (34.5)	3.6 ± 1.6	25.2 ± 3.4	9.1 ± 1.4
>60	128 (24.6)	3.3 ± 1.5	24.7 ± 3.5	9.2 ± 1.1
Education				
Primary	61 (11.7)	2.7 ± 1.5	23.8 ± 3.8	9.1 ± 1.6
Secondary	311 (59.7)	3.8 ± 1.6	25.8 ± 3.5	9.2 ± 1.1
University degree	126 (24.2)	4.3 ± 1.5	26.6 ± 3.2	9.2 ± 1.4
Illiterate	23 (4.4)	2.7 ± 1.3	23.7 ± 2.0	8.9 ± 1.5
Occupation				
Government sector	27 (5.2)	4.7 ± 1.2	27.6 ± 2.7	8.9 ± 1.7
Private sector	188 (36.1)	3.7 ± 1.6	25.9 ± 3.4	9.1 ± 1.4
Student	25 (4.8)	3.8 ± 1.5	25.9 ± 2.9	8.4 ± 1.2
Seasonal worker	16 (3.1)	3.2 ± 1.9	24.2 ± 1.6	8.4 ± 1.9
Retired	46 (8.8)	3.6 ± 1.5	25.5 ± 2.7	9.4 ± 0.9
Housewife	160 (30.7)	3.8 ± 1.7	25.5 ± 3.2	9.4 ± 0.9
Unemployed	59 (11.3)	3.2 ± 1.2	23.4 ± 5.2	9.2 ± 1.0

**Table 2:** Knowledge regarding dengue agent, symptom, transmission and treatment.

Study Population	Mantrijeron *N* = 257	Demangan *N* = 264	Total *N* = 521
Knowledge Item	Correct Answer N (%)		
Dengue, agent, symptom, transmission, treatment			
→ DHF is an abbreviation for Dengue Haemorrhagic Fever	93 (36.2)	109 (41.3)	202 (38.8)
→ Using repellent in the morning till evening is one way to prevent dengue	72 (28.0)	71 (26.9)	143 (27.4)
→ Having several days of high fever is one dengue symptom	71 (27.6)	72 (27.3)	143 (27.4)
→ Paracetamol and sponging with tepid water are types of first aid when infected with dengue.	72 (28.0)	65 (24.6)	137 (26.3)
→ Discarded material and bathtubs are potential *Aedes aegypti* breeding sites inside the house.	156 (60.7)	145 (54.9)	301 (57.8)
→ Aedes *aegypti* biting time is in the morning till evening	99 (38.5)	96 (36.4)	195 (37.4)
→ Dengue cannot be transmitted by direct contact with a dengue patient	210 (81.7)	216 (81.8)	426 (81.8)
→ Ditches are not potential breeding sites for *Aedes*	75 (29.2)	72 (27.3)	147 (28.2)

**Table 3:** Attitude towards Dengue Fever (DF) prevention and treatment.

Study Population	Mantrijeron *N* = 257	Demangan *N* = 264	Total *N* = 521
Attitude Statement	*N* (%)		
I don’t bother with the larvae in the indoor water container			
Strongly agree	7 (2.7)	6 (2.3)	13 (2.5)
Agree	4 (1.6)	11 (4.2)	15 (2.9)
Disagree	125 (48.6)	129 (48.9)	254 (48.8)
Strongly disagree	120 (46.7)	116 (43.9)	236 (45.3)
Don’t know	1 (0.4)	2 (0.8)	3 (0.6)
I need to take my family to a hospital immediately if infected by DF			
Strongly agree	143 (55.6)	131 (49.6)	274 (52.6)
Agree	110 (42.8)	121 (45.8)	231 (44.3)
Disagree	1 (0.4)	6 (2.3)	7 (1.3)
Strongly disagree	2 (0.8)	4 (1.5)	6 (1.2)
Don’t know	1 (0.4)	2 (0.8)	3 (0.6)
It is not necessary to clean up the bathtub routinely if not dirty			
Strongly agree	4 (1.6)	4 (1.5)	8 (1.5)
Agree	32 (12.5)	43 (16.3)	72 (14.4)
Disagree	164 (63.8)	166 (62.9)	330 (63.3)
Strongly disagree	56 (21.8)	49 (18.6)	105 (20.2)
Don’t know	1 (0.4)	2 (0.8)	3 (0.6)
It is necessary to brush bathtubs to eliminate mosquito eggs			
Strongly agree	108 (42.0)	112 (42.4)	220 (42.2)
Agree	133 (51.8)	138 (52.3)	271 (52.0)
Disagree	6 (2.3)	4 (1.5)	10 (1.9)
Strongly disagree	8 (3.1)	8 (3.0)	16 (3.1)
Don’t know	2 (0.8)	2 (0.8)	4 (0.8)
I will leave the unused plastic mineral water cans outside my house			
Strongly agree	4 (1.6)	5 (1.9)	9 (1.7)
Agree	9 (3.5)	14 (5.3)	23 (4.4)
Disagree	149 (58.0)	148 (56.1)	297 (57.0)
Strongly disagree	93 (36.2)	95 (36.0)	188 (36.1)
Don’t know	2 (0.8)	2 (0.8)	4 (0.8)
I don’t need to monitor larvae in my environment			
Strongly agree	3 (1.2)	3 (1.1)	6 (1.2)
Agree	22 (8.6)	31 (11.7)	53 (10.2)
Disagree	160 (62.3)	168 (63.6)	328 (63.0)
Strongly disagree	66 (25.7)	59 (22.3)	125 (24.0)
Don’t know	6 (2.3)	3 (1.1)	9 (1.7)
If I have a fever for 3 consecutive days without any other symptoms (influenza, cough, diarrhea), I suspect that I have dengue fever			
Strongly agree	46 (17.9)	49 (18.6)	95 (18.2)
Agree	182 (70.5)	174 (65.9)	356 (68.3)
Disagree	23 (8.9)	34 (12.9)	57 (10.9)
Strongly disagree	4 (1.6)	3 (1.1)	7 (1.3)
Don’t know	2 (0.8)	4 (1.5)	6 (1.2)
In my opinion, everyone has the same risk to get infected by dengue fever			
Strongly agree	73 (28.4)	80 (30.3)	153 (29.4)
Agree	161 (62.6)	160 (60.6)	321 (61.6)
Disagree	19 (7.4)	14 (5.3)	33 (6.3)
Strongly disagree	2 (0.8)	6 (2.3)	8 (1.5)
Don’t know	2 (0.8)	4 (1.5)	6 (1.2)

**Table 4:** Preventive measure regarding Dengue Fever prevention.

Study Population	Mantrijeron *N* = 257	Demangan *N* = 264	Total *N* = 521
Practice Items	Good Practice *N* (%)		
→ I pay attention to existing larvae in indoor water containers	238 (92.2)	248 (93.2)	486 (92.7)
→ I clean and brush water containers if any larvae inside	236 (91.5)	251 (94.4)	487 (92.9)
→ I clean my containers one to three times a week	239 (92.6)	242 (91.0)	481 (91.8)
→ I always keep water containers in my house closed	174 (67.4)	166 (62.4)	340 (64.9)
→ I cover or recycle discarded material outside the house	255 (98.8)	258 (97.0)	513 (97.9)
→ All my family members are responsible for cleaning water container	254 (98.4)	262 (98.5)	516 (98.5)
→ I clean water containers by draining and brushing	246 (95.3)	259 (97.4)	505 (96.4)
→ I use mosquito repellent	161 (62.4)	160 (60.2)	321 (61.3)
→ I use repellent or mosquito coil or mosquito spray in the morning and evening	60 (23.3)	53 (19.9)	113 (21.6)

**Table 5:** Effects of Control Card Intervention to research group (intervention and control group).

Variable	Effect of Control Card Intervention	
	IRR (95% CI)	*p*-Value
Number of containers positive with larvae	1.71 (0.87–3.36)	0.11
Number of houses positive with larvae	1.42 (0.69–2.92)	0.33
